# Granulocytic sarcoma of the pancreas: A case report and review of the literatures

**DOI:** 10.1186/1471-230X-10-80

**Published:** 2010-07-12

**Authors:** Yefei Rong, Dansong Wang, Wenhui Lou, Tiantao Kuang, Dayong Jin

**Affiliations:** 1 Pancreatic Cancer Group, Department of General Surgery, Zhongshan Hospital, Fudan University, Shanghai, China, 200032

## Abstract

**Background:**

Granulocytic sarcoma (GS) is a form of acute myeloid leukemia (AML), also known as extramedullary myeloid tumor or chloroma. It forms a solid malignant tumor consisting of myelocytes or granulocytes and is typically located in bone while occurrence in other parts of the body is rare.

**Case presentation:**

We reported a 40-year-old male patient who had jaundice, highly elevated bilirubin, and a mass highly suspicious of pancreatic head carcinoma. We performed surgery and the pathology and immunohistochemistry suggested GS; however the blood test and the bone marrow infiltration showed no evidence of AML. In our review of the published reports of GS, we only found six reports of the GS in the pancreas, and we suggested that immunohistochemical staining should be used to accurately differentiate GS from other pancreatic cancer and other types of leukemia.

**Conclusions:**

The accurate diagnosis of GS is necessary for determining prognosis and deciding appropriate therapy.

## Background

Granulocytic sarcoma (GS), also called extramedulary myeloblastoma or chloroma, is a rare solid tumor composed of immature myeloid cells [1]. The tumor may precede or occur concurrently with acute or chronic myeloid leukaemia or less often with polycythemia vera and primary myelofibrosis. Granulocytic sarcomas usually occurred in skin, soft tissue, lymph nodes, and bones [2]. Only few cases of GS of the pancreas had been reported in literatures [3,4]. We hereby reported a case of GS in the pancreas, in a patient without AML.

## Case presentation

The patient was a 40-year-old man. He had no significant past medical history. He initially went to the outside hospital on Jan 20, 2009, with the symptom of jaundice and weight loss of 5 Kg in one month. He described that his skin and sclera got yellow and gradually got worse in one month. He denied fever, chills, nausea, or vomiting. His physical examination at the time was only yellow skin and sclera. His routine laboratory test only showed the elevated total bilirubin and direct bilirubin. MRCP of the other hospital showed the malignant tumor in the distal common bile duct.

**Figure 1:**
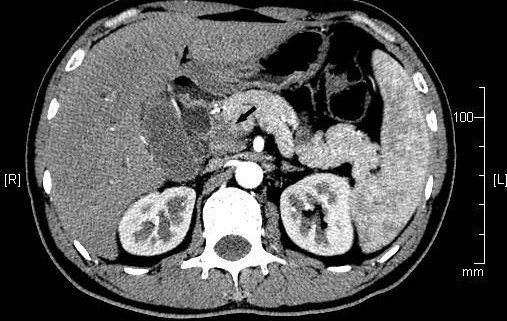
**Abdominal computed tomography**. Abdominal computed tomography showed a low density mass at the pancreatic head (arrows).

He was referred to our department for surgical treatment on Jan 24, 2009. At this time, his symptoms and physical examination results remained unchanged. Hemoglobin: 118g/L, white blood cell count: 3,600/mm^3^, platelets: 142 × 10^3^/mm^3^, AST: 114U/L, ALT: 250U/L, total bilirubin: 43.2 μmol/L, direct bilirubin: 35.2 μmol/L, serum creatinine:80 μmol/L,AFP:1.7ng/L,CA19-9:13.1U/L (normal < 37U/L). A CT scan (performed as a three-dimensional multi-detector scan) revealed a low-density mass at the pancreatic head (Figure 1). There was no evidence of ascites, and all of the peri-hepatic and peri-pancreatic visceral vessels were not invaded. Exploratory laparotomy was performed because pancreatic cancer was highly suspected.

**Figure 2:**
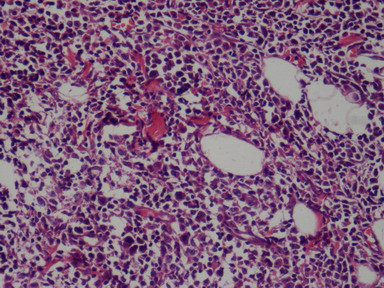
**Hematoxylin-eosin staining of the pancreatic GS**. HE staining showed the infiltration of pancreas by immature neoplastic myeloid cells.

**Figure 3:**
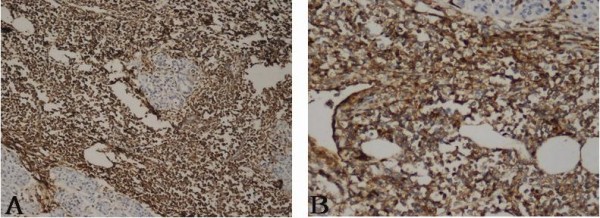
**Immunohistochemistry of the pancreatic GS**. (A) immunohistochemical detection of myeloperoxidase. Myeloid cells show strong positive signal; (B) immunohistochemical detection of CD43. Myeloid cells show strong positive signal.

At abdominal exploration on Feb 5, 2009, a mass was found in the pancreatic head. So we performed Whipple surgery. All pathology specimens were routinely processed. Histological examination showed diffusely infiltrating mono-morphous population of immature blast-like cells. The cells were intermediate in size and round to oval in shape with mild to moderate basophilic cytoplasm without granules (Figure 2). Immunohistochemical stains were performed on the paraffin-embedded sections. The tumor cells demonstrated positive reaction to myeloperoxidase (MPO), CD43 antibodies and negative to CD20 and CD2 monoclonal antibodies (Figure 3). Taking these results together, a diagnosis of GS was rendered. When the pathological results were confirmed; we performed the bone marrow infiltration. There was no evidence of AML. The patient was discharged on postoperative day12 in stable condition. Although the patient had no evidence of concomitant AML, the haemologist did suggest chemotherapy with cytarabine. The patient is followed up and currently doing well. The blood test and bone marrow infiltration show no evidence of AML.

## Discussions and Conclusions

Since 1966, about 1,000 cases of GS located outside bone had been published. GS of the pancreas was extremely rare; and only few cases were previously described. Several chromosome rearrangements were associated with GS, especially t (8; 21) and, less often inv (16) (p13; q22) [5-7]. Both rearrangements involved the core binding factor (CBF) gene and associated with high rates of complete remission and long-term disease-free survival in AML [8,9].

In the differential diagnosis, the most important diseases to be considered were non-Hodgkin lymphomas of the lymhoblastic type, Burkitt lymphoma, large-cell lymphoma and small round cell tumors [10]. Diagnostic confirmation of GS usually required immunohistochemical stainings for expression of myeloid associated enzymes. In addition to MPO, lysozyme, and chloroacetate esterase, GS usually expressed myeloid-associated antigens such as CD43, CD13, CD33, and CD117, but were negative with lymphoid antigens such as CD3 and CD20.Traweek et al[11] found that the use of an immunohistochemical panel including CD20, CD43, CD68, and MPO could successfully identify the vast majority of GS (90%).

For the treatment of the GS, radiation therapy or surgical resection had been found less effective than chemotherapy on improving the disease-free interval or disease-free survival [2,3]. Therefore it was very important for correct diagnosis of GS. As for GS might occur simultaneously with leukemia, the mass in these patients should be suspicious of GS and perhaps the fine needle biopsy should be performed before surgery or radiation therapy. However some patients with GS had no concomitant symptoms of leukemia, these patients were initially misdiagnosed. Nonleukemic patients who present with GS, most will develop AML within 1 year (median 5 months). For patients with known myeloproliferative disorders, the development of GS was a strongly negative prognostic factor for AML. Overall, the median survival was 7-20 months after the diagnosis of GS [12,13]. Once the diagnosis of GS had been confirmed by pathology and immunohistochemistry, the patients should be treated with chemotherapy even for the patients who had no concomitant leukemia.

## Consent

The patient has given their consent for the case report to be published. Written informed consent was obtained from the patient for publication of this case report and any accompanying images. A copy of the written consent is available for review by the Editor-in-Chief of this journal.

## Competing interests

The authors have neither financial nor non-financial competing interests to declare in relation to this manuscript.

## Authors' contributions

All authors read and approved the final manuscript. YFR and DYJ: Operating team and drafted the manuscript; DSW, WHL and TTK: operating team, post-operating care and helped in drafting the final manuscript.

## Pre-publication history

The pre-publication history for this paper can be accessed here:

http://www.biomedcentral.com/1471-230X/10/80/prepub
